# Choice of Model for Estimation of Adsorption Isotherm Parameters in Gradient Elution Preparative Liquid Chromatography

**DOI:** 10.1007/s10337-015-2949-0

**Published:** 2015-07-25

**Authors:** Marek Leśko, Dennis Åsberg, Martin Enmark, Jörgen Samuelsson, Torgny Fornstedt, Krzysztof Kaczmarski

**Affiliations:** Department of Chemical and Process Engineering, Rzeszów University of Technology, 35 959 Rzeszów, Poland; Department of Engineering and Chemical Sciences, INTERACT, Karlstad University, 651 88 Karlstad, Sweden

**Keywords:** Adsorption isotherm, Gradient elution, Inverse method, Overloaded profiles

## Abstract

The inverse method is a numerical method for fast estimation of adsorption isotherm parameters directly from a few overloaded elution profiles and it was recently extended to adsorption isotherm acquisition in gradient elution conditions. However, the inverse method in gradient elution is cumbersome due to the complex adsorption isotherm models found in gradient elution. In this case, physicochemically correct adsorption models have very long calculation times. The aim of this study is to investigate the possibility of using a less complex adsorption isotherm model, with fewer adjustable parameters, but with preserved/acceptable predictive abilities. We found that equal or better agreement between experimental and predicted elution profiles could be achieved with less complex models. By being able to select a model with fewer adjustable parameters, the calculation times can be reduced by at least a factor of 10.

## Introduction

Preparative liquid chromatography (LC) is an important separation technique that allows separation and purification of compounds from a few milligrams to many tons of pure substances per year [1]. Gradient elution is the most important programming technique and is used for separating compounds which have a wide range of retention times [2]. Gradient elution can save time and reduce solvent consumption by decreasing the run time [3, 4].

To take full advantage of gradient elution, the separation needs to be optimized. However, there are many factors which have to be considered when optimizing a process in gradient elution such as sample concentration, flow rate, amount of injected sample and gradient profile [5]. Empirical optimizing of one parameter at a time is time consuming and numerical optimization is therefore preferred. In numerical optimization, the adsorption isotherm for each component needs to be known [6]. Therefore, determination of the competitive adsorption isotherm model is essential [7].

The inverse method of chromatography [8, 9] is a method to acquire adsorption isotherms especially suited for process optimization, where calculated overloaded elution profiles are fitted to experimental ones. Recently, we extended the inverse method to acquisition of single component adsorption isotherms [10, 11] and competitive [12] in gradient elution. The extended inverse method only requires gradient elution experiments—i.e., no isocratic experiments are needed.

In previous studies [10–12], the adsorption isotherm model which was deemed most thermodynamically correct was used in the inverse method. This led to tedious and not straightforward calculations due to the complexity of the adsorption isotherms. The aim of this study is to evaluate the possibility of using a less complex adsorption isotherm model with fewer adjustable parameters, but with acceptable predictive abilities. Being able to use a model with fewer adjustable parameters will speed up the computations which is important when optimizing the separation process.

## Theory

The equilibrium-dispersive (ED) model [6] was applied to model the mass balance of the solutes in the chromatographic column. For component *i*, the mass balance of the ED model for an infinitesimal volume of adsorbent bed is:

1 $$ \frac{{\partial C_{i} }}{\partial t} + F\frac{{\partial q_{i} }}{\partial t} + \frac{u}{{\varepsilon_{t} }}\frac{{\partial C_{i} }}{\partial z} = D_{\text{a}} \frac{{\partial^{2} C_{i} }}{{\partial z^{2} }}, $$

where *C*_*i*_ and *q*_*i*_ are the solute concentration in the mobile and stationary phases, respectively, *F* is the phase ratio, *D*_a_ is the apparent axial dispersion coefficient, *ε*_*t*_ is the total porosity, *u* is the superficial velocity, *t* is the time coordinate and *z* is the axial coordinate. In gradient elution, the organic modifier is treated as an unretained component and is coupled with the solute mass balances through the adsorption isotherms. The coupled system of the mass balance equations, Eq. (1), was solved using the orthogonal collocation on finite elements method described in [13, 14]. In gradient elution it is usually assumed that the adsorption isotherm model does not change with the fraction of the modifier in the eluent, only the parameters in the adsorption isotherm model does [15–18].

The Langmuir adsorption isotherm incorporating a dependence on the organic modifier can be written as:

2 $$ q_{i} \left( {C_{1} ,C_{2} ,\varphi } \right) = \frac{{a_{i} {\text{e}}^{{ - S_{{a_{i} }} \varphi }} C_{i} }}{{1 + b_{1} {\text{e}}^{{ - S_{{b_{1} }} \varphi }} C_{1} + b_{2} {\text{e}}^{{ - S_{{b_{2} }} \varphi }} C_{2} }}, $$

where *C*_*i*_ and *q*_*i*_ are the solute concentration in the mobile and stationary phases, respectively, for component *i*, *φ* is the fraction of modifier, in this case methanol, in the eluent, and *S*_*a*_ and *S*_*b*_ are coefficients describing the modifier dependence of the constant (*a*) and the association equilibrium constant (*b*).

To obtain a thermodynamic consistent Langmuir model, the monolayer saturation capacity (*q*_*s*_) must be identical for components 1 and 2. In gradient elution, it becomes:

3 $$ q_{1} \left( {C_{1} ,C_{2} ,\varphi } \right) = \frac{{a_{{{\text{I}},1}} {\text{e}}^{{ - S_{{a_{{{\text{I}},1}} }} \varphi }} C_{1} }}{{1 + \frac{{a_{{{\text{I}},1}} }}{{q_{s,1} }}{\text{e}}^{{ - S_{{a_{{{\text{I}},1}} }} \varphi }} C_{1} + \frac{{a_{{{\text{I}},2}} }}{{q_{s,1} }}{\text{e}}^{{ - S_{{a_{{{\text{I}},2}} }} \varphi }} C_{2} }}, $$

where the monolayer saturation capacity is assumed to be independent of the methanol fraction. For the last eluting compound, an additional Langmuir term was needed to accurately model the experimental data:

4 $$ q_{2} \left( {C_{1} ,C_{2} ,\varphi } \right) = \frac{{a_{{{\text{I}},2}} {\text{e}}^{{ - S_{{a_{{{\text{I}},2}} }} \varphi }} C_{2} }}{{1 + \frac{{a_{{{\text{I}},1}} }}{{q_{s,1} }}{\text{e}}^{{ - S_{{a_{{{\text{I}},1}} }} \varphi }} C_{1} + \frac{{a_{{{\text{I}},2}} }}{{q_{s,1} }}{\text{e}}^{{ - S_{{a_{{{\text{I}},2}} }} \varphi }} C_{2} }} + \frac{{a_{{{\text{II}},2}} {\text{e}}^{{ - S_{{a_{{{\text{II}},2}} }} \varphi }} C_{2} }}{{1 + \frac{{a_{{{\text{II}},2}} }}{{q_{s,2} }}{\text{e}}^{{ - S_{{a_{{{\text{II}},2}} }} \varphi }} C_{2} }}, $$

where subscripts I and II denote adsorption sites with different adsorption energies. Site II is accessible only to the last eluting component cycloheptanone.

In a previous study, we assume that all adsorption sites are nonselective, so both solutes have access to them. Then we get the competitive bi-Langmuir model [6]:

5 $$ q_{i} \left( {C_{1} ,C_{2} ,\varphi } \right) = \frac{{a_{{{\text{I}},i}} {\text{e}}^{{ - S_{{a_{{{\text{I}},i}} }} \varphi }} C_{i} }}{{1 + b_{{{\text{I}},1}} {\text{e}}^{{ - S_{{b_{{{\text{I}},1}} }} \varphi }} C_{1} + b_{{{\text{I}},2}} {\text{e}}^{{ - S_{{b_{{{\text{I}},2}} }} \varphi }} C_{2} }} + \frac{{a_{{{\text{II}},i}} {\text{e}}^{{ - S_{{a_{{{\text{II}},i}} }} \varphi }} C_{i} }}{{1 + b_{{{\text{II}},1}} {\text{e}}^{{ - S_{{b_{{{\text{II}},1}} }} \varphi }} C_{1} + b_{{{\text{II}},2}} {\text{e}}^{{ - S_{{b_{{{\text{II}},2}} }} \varphi }} C_{2} }}. $$

The Tóth model [6] for the two-component case, extended to the gradient elution, can be written as:

6 $$ q_{i} \left( {C_{1} ,C_{2} ,\varphi } \right) = \frac{{a_{i} {\text{e}}^{{ - S_{{a_{i} }} \varphi }} C_{i} }}{{\left( {1 + \left( {b_{1} {\text{e}}^{{ - S_{{b_{1} }} \varphi }} C_{1} + b_{2} {\text{e}}^{{ - S_{{b_{2} }} \varphi }} C_{2} } \right)^{\nu } } \right)^{1/\nu } }}, $$

where *ν* is a parameter which characterizes the heterogeneity of the adsorbent surface. If *ν* = 1, the Tóth model becomes the Langmuir model. In this study, we assume that the heterogeneity of the adsorbent surface is independent of the methanol fraction.

## Materials and Method

The experimental system containing two components, cyclohexanone and cycloheptanone, has been described before [10, 12].

### Simulated Data

The simulated chromatographic elution profiles were obtained with a 150 × 4.6 mm column having a hold-up volume of 1.38 mL and the flow rate was 1 mL/min, while the column efficiency was 2000 plates. The competitive bi-Langmuir isotherm, Eq. (3), was used to generate the simulated data. Four different linear gradients were used with slopes 1, 2, 3 and 4 %/min and run from 24 to 56 % (v/v) methanol in the mobile phase. The sample concentration was 0.4 M of each of the two components and the injection volume was 400 μL.

### Estimation Procedure

Simultaneous estimation of all model parameters is hard if the starting guesses are not good because of the difficulty in finding the global minimum or other satisfactory solutions for the algorithm [10, 12]. Thus, the estimation from experimental data was done in steps where the goal of the first step was to find starting guesses to use in the last estimation step:

1. In the first step, the analytical version of the adsorption isotherm was considered, i.e., the *b*-parameters were equal to 0. Estimation was done separately for cyclohexanone and cycloheptanone from the retention times of analytical peaks obtained in gradient elution. This was done by minimizing the difference between the calculated and experimental retention times.
2. In the second step, initial guesses for *b* and *S*_*b*_ or *q*_*s*_ or *ν* were determined by solving the ED model with the fast, but less accurate Rouchon algorithm [19]. Two experimental peaks were used: the one obtained for highest sample concentration and steepest gradient slope (0.4 M, 4 %/min), and the one obtained for lowest sample concentration and lowest slope of gradient (0.1 M, 1 %/min).
3. In the last step, all parameters were estimated on the basis of the same concentration profiles as in step 2 with the OCFE method.

For the simulated data, the procedure described above could be simplified. Step 1 was the same as that described above, but steps 2 and 3 were combined and parameters *b* and *S*_*b*_ or *q*_*s*_ or *ν* were estimated based on the four overloaded peaks with concentration 400 mM obtained for gradient 1 and 4 %/min. The parameters obtained in the first step were kept constant with the OCFE method.

## Result and Discussion

The aim of this study is to investigate the possibility of applying different adsorption isotherm models to the same set of experimental data and comparing the model’s ability to predict overloaded elution profiles.

### Proof of Concept Using Simulated Data

The bi-Langmuir adsorption isotherm, Eq. (5), was chosen to generate a set of simulated elution profiles. This model was selected because it had the largest number of adjustable parameters among the investigated models and we were interested in evaluating if a less complex model could be used to describe the elution profiles generated by this model. The Tóth model, the Langmuir model, and the thermodynamically consistent Langmuir model were fitted to the simulated data. The estimated elution profiles from all three models were found to be in good agreement with the ones used as a basis for the estimation. The best agreement was obtained with the Tóth model with the average area overlaps [20] 97 and 99 % for components 1 and 2, respectively. The average area overlaps for the Langmuir model were equal to 97 and 94 % and for the thermodynamically consistent Langmuir model 96 and 93 % which were slightly worse, but still very good.

**Fig. 1:**
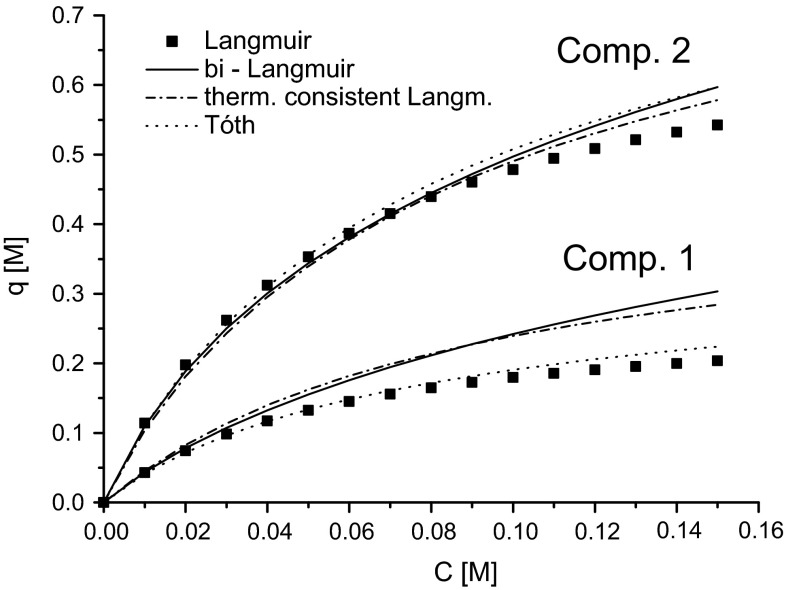
Comparison between the adsorption isotherms of the true bi-Langmuir model and the three models estimated from the simulated data, plotted at 35 % methanol fraction in the eluent. The concentration ratio for components 1 and 2 is 1:1

The shape of the original bi-Langmuir isotherm model used to generate the simulated elution profiles are compared to the estimated ones from the Langmuir, thermodynamically consistent Langmuir and the Tóth models in Fig. 1 at the 35 %-methanol plateau for a 1:1 ratio of components 1 and 2. For component 1, the agreement is very good for the thermodynamically consistent Langmuir model and fair for the Langmuir model and Tóth model. The deviation is largest at high concentrations. For component 2, all models have excellent agreement with the original bi-Langmuir isotherm model.

From these results, we can conclude that elution profiles generated with the bi-Langmuir model can be described by a less complex adsorption isotherm models. One must, however, stress that the simulated data did not contain any noise and therefore similar calculations are needed to be done using real experimental data instead.

### Calculations Using Experimental Data

**Fig. 2:**
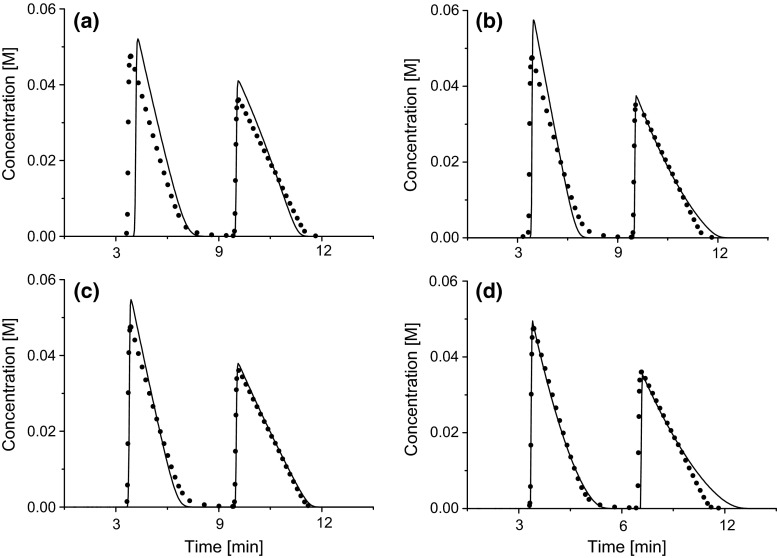
Comparison between the experimental (*symbols*) and predicted (*solid* *lines*) concentration profiles for experimental data of cyclohexanone (first eluting component) and cycloheptanone (second eluting component) corresponding to concentration 100 mM, gradient slope 3 %/min for **a** the Langmuir model, **b** the bi-Langmuir model, **c** the thermodynamically consistent Langmuir model and **d** the Tóth model

Four different adsorption models were fitted with the inverse method in gradient elution to the same experimental data. The agreement between experimental and predicted elution profiles is good for the used models at all gradient slopes and concentrations, as can be seen in Fig. 2. By calculating the area overlap between experimental and predicted elution profiles for all experimental conditions, it is possible to estimate which model best predicts the elution profiles.

The area overlap differs depending on the isotherm model used, sample concentration, and gradient slope, but in almost all cases greater than 80 % and in many cases exceeds 90 %. The isotherm model which gave the highest average area overlap for cyclohexanone was the Tóth model with an overlap equal to 95 %. The rest of the considered models had average area overlaps below 90 % for cyclohexanone. The highest average area overlap for cycloheptanone was obtained with the thermodynamically consistent Langmuir model with an overlap of 97 %, but all four models gave overlaps above 92 % for cycloheptanone. Seen with both components in mind, the Tóth model consistently gave the best average area overlaps. In Table 1, the average overlap is presented. The bi-Langmuir model, which in the previous studies [10, 12] was found to be the thermodynamically most correct model, had the worst agreement between the calculated and experimental elution profiles.

**Table 1:** Average area overlaps in % between the experimental and calculated elution profiles for cyclohexanone (C6) and cycloheptanone (C7) for the four investigated adsorption isotherm models

Langmuir		bi-Langmuir		Therm. consistent Langmuir		Tóth	
C6	C7	C6	C7	C6	C7	C6	C7
85.3	93.3	85.1	92.4	89.9	96.9	94.5	93.4

The average is from 16 experimental systems where the concentrations were 0.1, 0.2, 0.3 and 0.4 M of each component and the gradient slopes were 1, 2, 3 and 4 %/min

If the complexity of the model and the number of adjustable parameters are also considered, the Langmuir model is probably the best choice for process optimization because the area overlap is “good enough” for subsequent numerical optimization and, due to its simplicity, the calculation times will be much lower than for the other models.

## Conclusions

The choice of adsorption isotherm model in the inverse method when applied to gradient elution was investigated using both synthetic and experimental data. Comparing the results from the different adsorption isotherm models indicates that the most complex model may not always lead to the best fit between experimental and calculated elution profiles. We believe that this is due to the difficulties of estimating such a large number of parameters that models like the bi-Langmuir have. The Langmuir model and thermodynamically consistent Langmuir model have 8 parameters, while the Tóth model and bi-Langmuir model have 9 and 16 parameters, respectively. Estimation of a smaller number of parameters is easier and faster. Therefore, rather counterintuitively, our results suggest selecting a model with fewer adjustable parameters in order to get the model with the best overall predictive power.

Two practical consequences are that (1) the inverse method used in gradient elution does not allow for the determination of a unique, unambiguous adsorption isotherm model and (2) a model with more adjustable parameters does not give a better agreement with the experimental data with the presented methodology. The first point is not a problem from an engineering point of view, because the only reasonable goal for using the inverse method is correct prediction of elution profiles for subsequent use in process optimization. The second point is probably due to the difficulties of estimating functions with a large number of adjustable parameters from chromatographic elution profiles.
